# Assessment of bevacizumab resistance increased by expression of BCAT1 in IDH1 wild-type glioblastoma: application of DSC perfusion MR imaging

**DOI:** 10.18632/oncotarget.11901

**Published:** 2016-09-08

**Authors:** Hye Rim Cho, Bora Hong, Hyeonjin Kim, Chul-Kee Park, Sung-Hye Park, Sunghyouk Park, Seung Hong Choi

**Affiliations:** ^1^ Department of Radiology, Seoul National University Hospital, Seoul, Korea; ^2^ Center for Nanoparticle Research, Institute for Basic Science (IBS), Seoul, Korea; ^3^ Department of Neurosurgery, Seoul National University Hospital, Seoul, Korea; ^4^ Department of Pathology, Seoul National University Hospital, Seoul, Korea; ^5^ College of Pharmacy, Natural Product Research Institute, Seoul National University, Seoul, Korea

**Keywords:** BCAT1, dynamic susceptibility contrast (DSC), bevacizumab, glioblastoma

## Abstract

BCAT1 (branched-chain amino acid trasaminase1) expression is necessary for the progression of IDH1 wild-type (WT) glioblastoma multiforme (GBM), which is known to be associated with aggressive tumors. The purpose of our study is to investigate the bevacizumab resistance increased by the expression of BCAT1 in IDH1 WT GBM in a rat model, which was evaluated using DSC perfusion MRI. BCAT1 sh#1 inhibits cell proliferation and limits cell migration potential *in vitro*. *In vivo* MRI showed that the increase in both tumor volume and nCBV after bevacizumab treatment in IDH1 WT tumors was significantly higher compared with BCAT1 sh#1tumors. In a histological analysis, more micro-vessel reformation by bevacizumab resistance was observed in IDH1 WT tumors than BCAT1 sh#1 tumors. These findings indicate that BCAT1 expression in IDH1 WT GBM increases resistance to bevacizumab treatment, which could be assessed by DSC perfusion MRI, and that nCBV can be a surrogate imaging biomarker for the prediction of antiangiogenic treatment in GBM.

## INTRODUCTION

Glioblastoma multiforme (GBM) is the most common malignant primary brain tumor in adults. Although the tumor is generally treated with surgical resection, chemotherapy, and radiation, recurrence is common, and the mean progression-free survival is just over 6 months [1]. Thus, there has been progress in understanding the molecular basis of the tumor's heterogeneity and aggressiveness. Various molecular sub-classifications have been proposed based on the genetic makeup of these tumors, with the hope that a better understanding of the origin of tumor cells and molecular pathogenesis may allow the prediction of the response to targeted therapies [2–5].

Among various pathways, angiogenesis, an essential feature of brain tumors, has become a target for multiple novel therapies [1, 6–8]. Interactions between tumor cells and blood vessels seem to facilitate tumor growth. Indeed, bevacizumab (Avastin; Genentech, South San Francisco, Calif), which is an antiangiogenic monoclonal antibody that binds vascular endothelial growth factor (VEGF), has been shown to increase progression-free survival in patients with newly diagnosed or recurrent GBM, presumably by inhibiting both the formation of vessels dependent on VEGF and the vascular permeability of these highly vascular tumors [1, 7, 9, 10].

BCAT1 (branched-chain amino acid trasaminase1) is a cytosolic enzyme that catalyzes the catabolism of branched-chain L-amino acids (BCAA) into branched-chain a-ketoacids (BCKA), while concomitantly converting a-KG to glutamate [11, 12]. Recently, Tönjes *et al*. [13] found that isocitrate dehydrogenase 1 (IDH1) WT-expressing GBMs have an increased expression of BCAT1, and they showed that expression of this enzyme is necessary for tumor growth and disease progression. Another study reported [11] that the expression of BCAT1 was significantly reduced in IDH1 MT glioma cells compared with their wild-type counterparts, and it was investigated by hyperpolarized ^13^C magnetic resonance spectroscopy (MRS). In addition, previous studies have shown that BCAT1 could serve as a novel target for GBM treatment [14]. Therefore, noninvasive assessment of BCAT1 activity could help confine the diagnosis and monitoring of tumors harboring the IDH1 WT and aid in the development and monitoring of BCAT1-targeting therapeutic efficacy [11, 13, 14].

Dynamic susceptibility contrast (DSC) perfusion MRI has been widely used as a diagnostic and prognostic tool in the clinical field, and it has shown utility for characterizing histopathological features and differentiating brain abnormalities, [15] investigating the prognosis of glioma patients [16–19] based on grade of gliomas, [16, 19, 20] predicting malignant transformation, recurrence or progression [21] and differentiating recurrent tumor from chemotherapy or radiation induced injury [22–24].

The expression of BCAT1 has been previously investigated using hyperpolarized ^13^C MRS [11], but it has not, to our knowledge, been studied with MRI perfusion techniques. Here, given the known role of BCAT1 in tumor cell proliferation and invasiveness [13], we investigate the bevacizumab resistance increased by BCAT1 expression in IDH1 WT GBM rat models using DSC perfusion MRI.

## RESULTS

### Characterization of BCAT1 knockdown IDH1 WT cell and tumor

**Figure 1:**
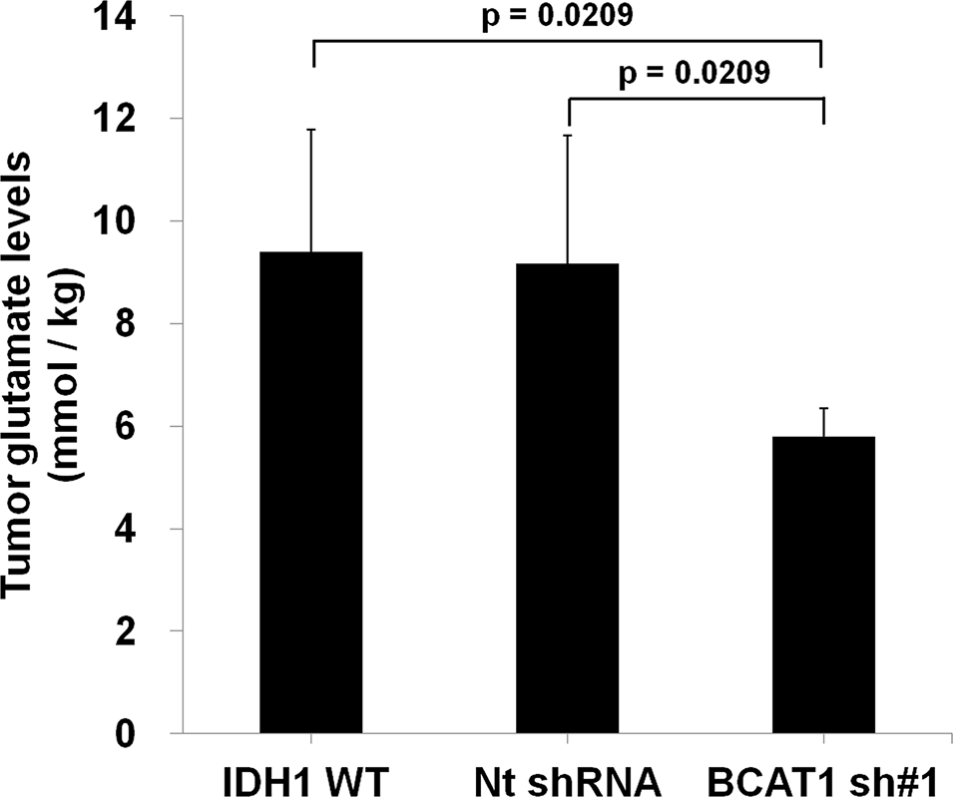
Analysis of tumor glutamate level of rat GBM model (*n =* 4 in each group) by MR spectroscopy The glutamate level, which was normalized by water, of BCAT1 sh#1 GBM decreased compare to IDH1 WT or Nt shRNA tumors in the rat GBM model (IDH1 WT: 10.17 mmol/kg; Nt shRNA: 9.29 mmol/kg; BCAT1 sh#1 : 5.90 mmol/kg, respectively).

**Figure 2:**
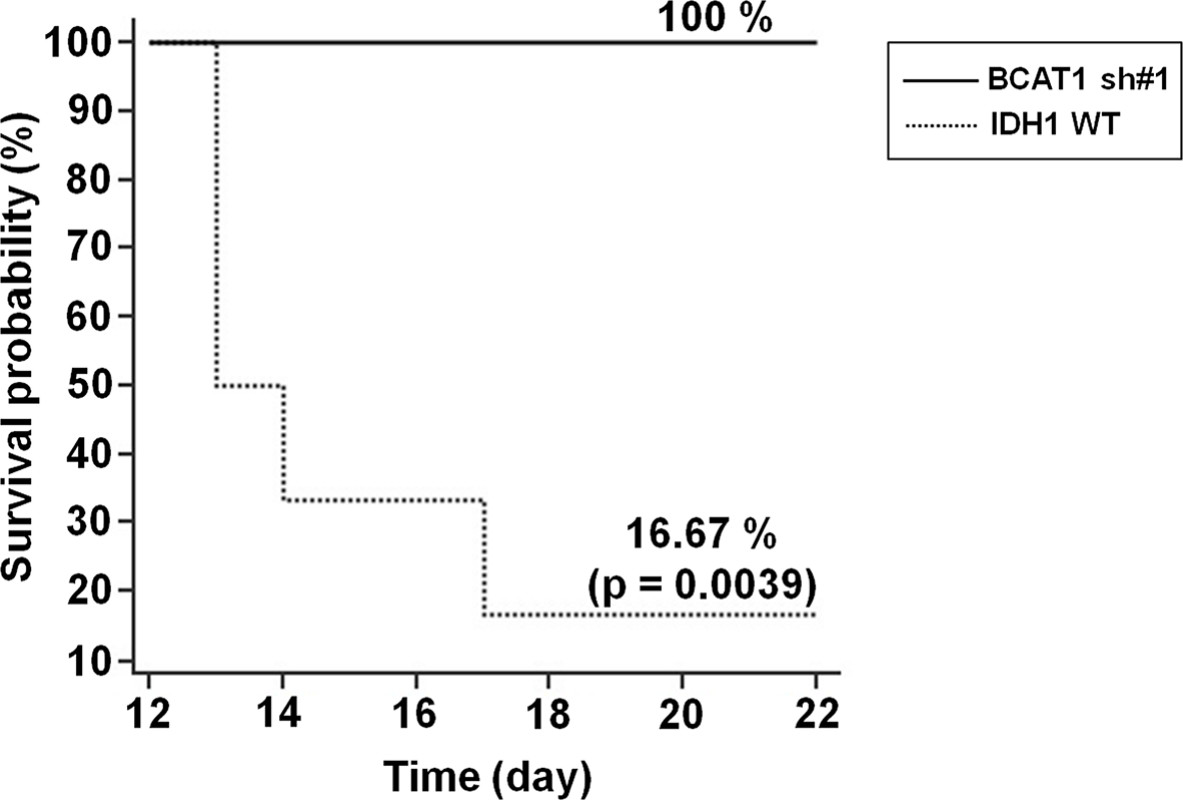
Survival rate of GBM rat models Survival was assessed every 24 hours for 22 days. As shown, a highly significant decreased survival rate was observed in IDH1 WT rats (*n* = 5) compared with BCAT1 sh#1 rats (*n* = 5) (*p* = 0.0039).

To investigate the role of BCAT1 in tumor cells, we used the IDH1 WT-expressing human U87 MG GBM cell line and stably knocked down BCAT1 using shRNA (Supplementary Figure S1 and S2). Proliferation and migration of BCAT1 sh#1 cells were significantly slower than IDH1 WT cells (Supplementary Figure S3A and S3B). Moreover, the shape of BCAT1 sh#1 cells showed a less extended and rounded appearance compare to IDH1 WT and Nt shRNA cells (Supplementary Figure S3C). Roundness was significantly lower in IDH1 WT cells than BCAT1 sh#1 cells (*p* = 0.0025) (Supplementary Figure S3D). To determine whether differential expressions of BCAT1 in IDH1 WT cells inhibit the glutamate-releasing metabolism, we measured the concentration of glutamate at both the intra- and extra- cellular levels. The decreased glutamate concentration was revealed in BCAT1 sh#1 cells compare to IDH1 WT cells in both intracellular (*p* = 0.0039) and extracellular level (*p* = 0.0039) (Supplementary Figure S3E and S3F). To extend this analysis, we measured decreased the glutamate level by BCAT1 sh#1 using MRS in the rat GBM model. The glutamate concentration was lower in the BCAT1 sh#1 GBM compared to the IDH1 WT and Nt shRNA (Figure 1). Furthermore, in terms of survival, the survival rate of the IDH1 WT group significantly decreased (*p* = 0.0039) compared with the BCAT1 sh#1 group (Figure 2). These observations suggest that IDH1 WT-expressing cell proliferation and tumor formation, which were reported in previous study [13], were enhanced by BCAT1 expression.

### Bevacizumab resistance evaluation using DSC perfusion MRI in rat GBM model

**Figure 3:**
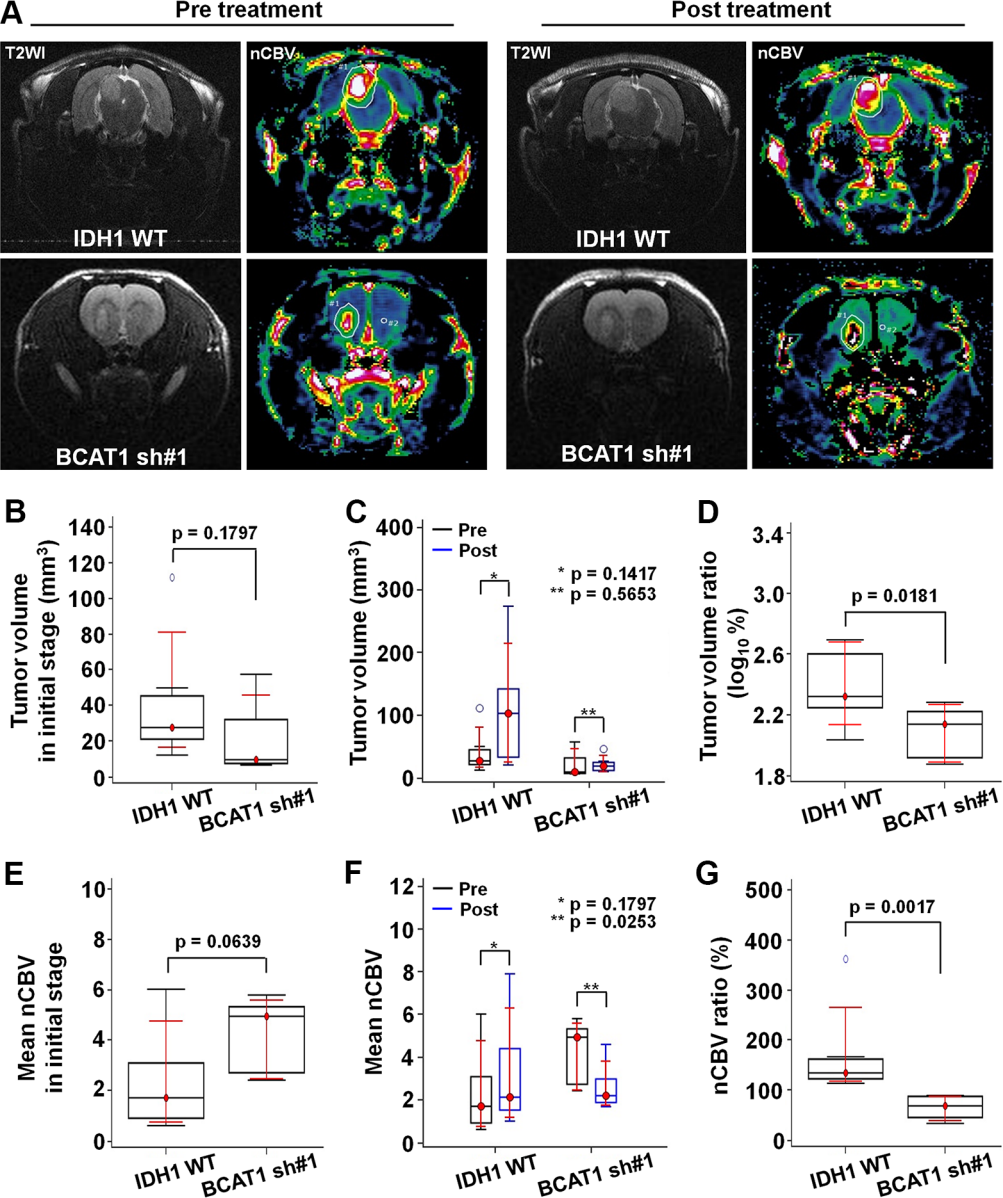
*In vivo* MRI (**A**) Tumor resistance to the bevacizumab is evaluated by DSC perfusion MRI. T2WI show tumor extents, and nCBV maps reveal the nCBV of the tumors (Upper: IDH1 WT, lower: BCAT1 sh#1). (**B**) The tumor volume in the BCAT1 sh#1 group is smaller than that in the IDH1 WT group at the pre-treatment stage without statistical significance (*p* = 0.1797). (**C**) A significant difference in tumor volume was not observed between pre- and post-treatment MRI in both IDH1 WT and BCAT1 sh#1 groups. (**D**) However, tumor volume ratio (log_10_%) was significantly increased in IDH1 WT group after bevacizumab treatment than BCAT1 sh#1 group (*p* = 0.0181). (**E**) No significant difference of nCBV was observed between IDH1 WT and BCAT1 sh#1 groups at initial stage (*p* = 0.0639) (**F**) The nCBV of IDH1 WT tumors increases after before bevacizumab treatment. However, all BCAT1 sh#1 tumors show a significant decrease in nCBV (*p* = 0.0253). (**G**) The nCBV ratio between pre- and post-treatment CBV maps is significantly higher in IDH1 WT tumors than BCAT1 sh#1 tumors (*p* = 0.0017).

To ascertain the resistance to the anti-angiogenic treatment in the GBM rat model, we obtained DSC perfusion MRI by using a 9.4T MR scanner and measured the CBV of the tumors. Figure 3 shows anatomical T2WIs and nCBV maps before and after bevacizumab treatment. Before the treatment, the tumor volume of BCAT1 sh#1 rats was smaller than IDH1 WT rats at initial without statistical significance (9.64 mm^3^ [IQR, 7.70–32.28] vs 27.77 mm^3^ [21.12–45.15]; *p* = 0.1797) (Figure 3B). The tumor volume increased after bevacizumab treatment in both IDH1 WT and BCAT1 sh#1 rats. However a significant difference was not observed between pre and post-treatment images in both groups (Figure 3C). Tumor volume ratio (log_10_%) in IDH1 WT was significantly increased after bevacizumab treatment than BCAT1 sh#1 rats (2.3210 [IQR, 2.2505–2.6005] vs 2.1370 [1.9173–2.2232]; *p* = 0.0181) (Figure 3D). The post-treatment DSC perfusion MRI of the IDH1 WT rats showed that nCBV increased after bevacizumab treatment. Post-treatment DSC perfusion MRI of the BCAT1 sh#1 rats showed a greater decrease in nCBV compare with IDH1 WT rats (Figure 3E and 3F). In contrast, BCAT1 sh#1 rats injected with saline instead of bevacizumab showed increased nCBV at post-treatment DSC perfusion MRI (Supplementary Figure S4). Moreover, a significant difference in nCBV ratio was observed between IDH1 WT and BCAT1 sh#1 rats (134.20% [IQR, 122.67–161.81] vs 68.88% [45.38–86.81]; *p* = 0.0017) (Figure 3G).

We evaluated off-target effect of shRNA by using a second shRNA (BCAT1 sh#3), which reveals which revealed similar change to IDH WT tumors in terms of both tumor volume and nCBV (Supplementary Figure S5).

### Histological analysis

**Figure 4:**
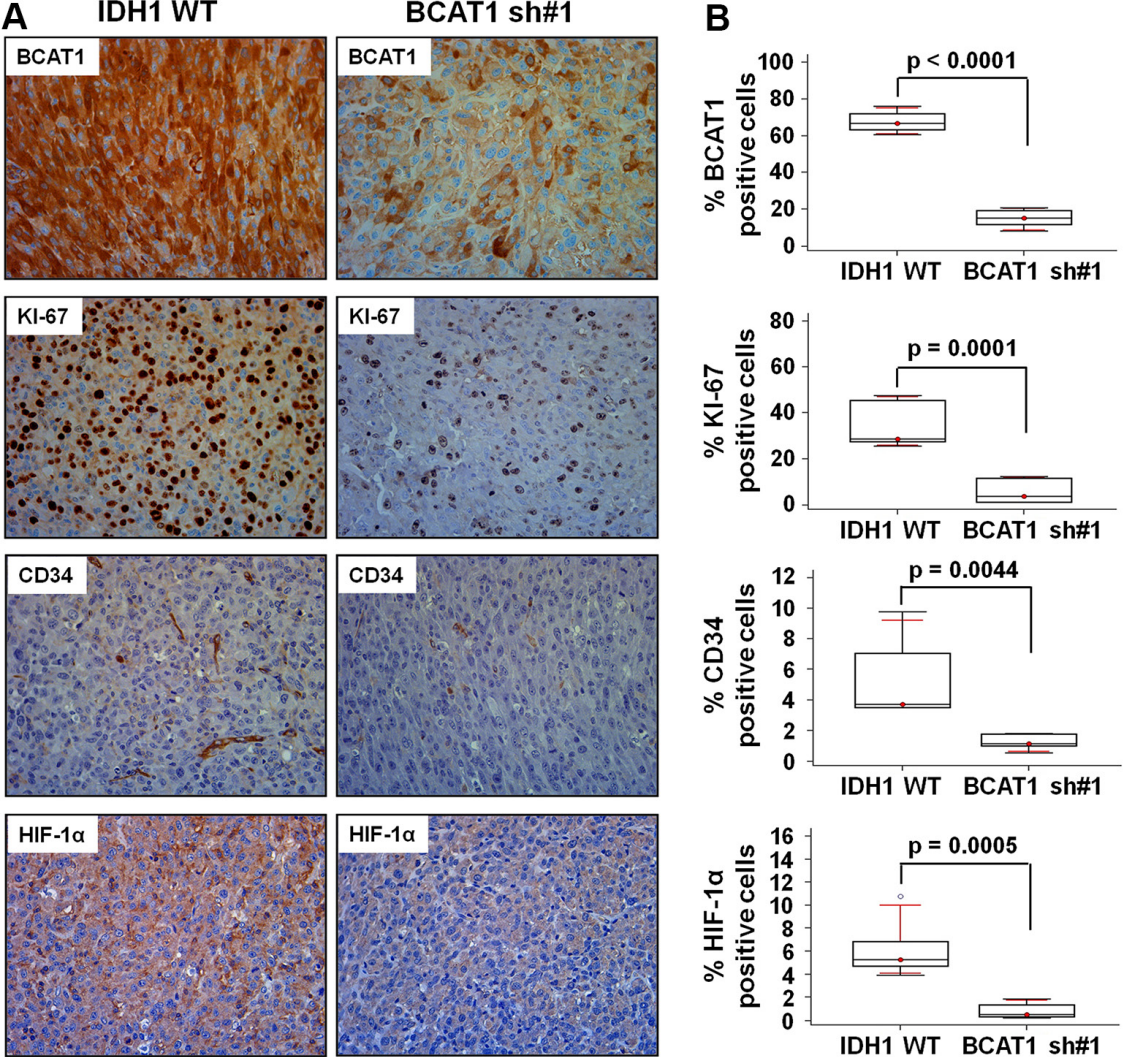
Histology analysis Immunohistochemistry images showing differential expression of BCAT1, KI-67, CD34, and HIF-1α from the IDH1 WT and BCAT1 sh#1 tumors. The first row shows a decreased BCAT1 level in BCAT1 sh#1 tissue. The rate of cell proliferation evaluated by KI-67 (second row), vascularity by CD34 (third row), and HIF-1a expression level (forth row) are decreased in BCAT1 sh#1 tumors. The expression is evaluated by DAB and shows the original magnification of ×40.

To further examine the effect of BCAT1 after bevacizumab treatment, we performed a histological analysis (Figure 4). The level of BCAT1 was lower in BCAT1 sh#1 rats than in IDH1 WT rats (14.99% [IQR, 9.65–20.33] vs 67.51% [61.59–73.42]; *p* < 0.0001). The tumor cell proliferation and vascularity, which were evaluated by KI-67 and CD34, respectively, were markedly decreased in BCAT1 sh#1 rats than IDH1 WT rats (5.48% [IQR,–0.04–11.00] vs 33.86% [23.56–44.18]; *p* = 0.0001, and 1.22% [IQR, 0.71–1.72] vs 5.20% [2.44–7.95]; *p* = 0.0044, respectively). Moreover, HIF-1α was expressed less in BCAT1 sh#1 rats (0.79% [IQR, 0.09–1.47] vs 6.13% [3.53–8.72]; *p* = 0.0005). These observations correlate with nCBV, which was obtained from DSC perfusion MRI, and suggest that the tumor resistance to the anti-angiogenic agent in IDH1 WT GBM is related to BCAT1.

## DISCUSSION

GBM, the most aggressive subtype of glioma, has high vascularization and diverse genetic alterations [1, 25, 26]. Given the dismal prognosis of GBM, it would be advantageous to identify early biomarkers of a response to therapy to avoid continuing ineffective treatments and to initiate other therapeutic strategies [1, 27, 28]. Furthermore, several altered genes, including TP53, PTEN, CDKN2A and EGFR, tend to occur in a defined order during the progression to a high-grade tumor. Among them, IDH1 mutations have been reported to be the most important for prognosis appeared in approximately 12% of GBMs, mostly secondary types with a good prognosis [29]. However, patients with IDH1 WT GBMs are much more, and have worse prognosis than those with IDH1 mutation, [30] so there have been efforts to find a biomarker to predict prognosis or improve treatment response of IDH1 WT GBMs. In the present study, we established BCAT1 sh#1 with IDH1 WT-expressing U87 MG cell lines and characterized according to previous report [13]. In addition, we applied the DSC perfusion MRI in a rat GBM model for the investigation of bevacizumab resistance increased by BCAT1 expression as a new finding.

In an initial cell study, BCAT1 sh#1 cells decreased more significantly in proliferation, migration, glutamate concentration than did IDH1 WT cells. Moreover, the cellular morphology showed a less extended and rounded appearance compare to IDH1 WT. Importantly, several previous studies have shown that the expression of BCAT1 is necessary for the progression of IDH1 WT gliomas, which sustain an aggressive growth phenotype, [13, 31] and our data are in line with previous reports [11, 31]. The first crucial insight gleaned from our data is that the different nCBV were investigated between IDH1 WT and BCAT1 sh#1 rats after bevacizumab treatment in an animal DSC perfusion MR study; nCBV increased in IDH1 WT tumors after bevacizumab treatment, but decreased in BCAT1 sh#1 rats. Moreover, BCAT1 sh#3 tumors to assess off-target effects also showed increased nCBV after bevacizumab treatment, which was similar to IDH1 WT tumors (Supplementary Figure S5). The bevacizumab is a recombinant humanized monoclonal antibody that binds to human VEGF-A [7, 32]. Particularly in GBM, the angiogenesis inhibitor bevacizumab has been shown to increase progression-free survival in patients with newly diagnosed or recurrent GBM, presumably by inhibiting both the formation of vessels dependent on VEGF and the vascular permeability of these highly vascular tumors [1, 33]. Nevertheless, nCBV of IDH1 WT rats increased after bevacizumab treatment, which seems to be correlated to excreted glutamate by BCAT1 activity fueled to the tumor cell for proliferation, suggesting that it induces resistance. BCAT1 sh#1, in contrast, blocked the BCAA catabolism by interfering with tumor energy production and macromolecule synthesis, and accommodated anti-angiogenic therapy by reducing tumor glutamate excretion [11–13]. These observations were further investigated by histological analysis. The expression levels of KI-67, CD34 and HIF-1α decreased more significantly after bevacizumab treatment in BCAT1 sh#1 rats than in IDH1 WT rats. These results suggest that the tumor resistance to the anti-angiogenic agent in IDH1 WT GBM is increased by BCAT1 expression. Associated with this phenomenon, our results also showed significantly lower growth in BCAT1 sh#1 tumors after treatment compared to IDH1 WT tumors (Figure 3), which might be affected by the initial tumor volume, but we believe that this factor was not significant, because the initial tumor volumes were not significantly different in a rat model. Similarly, a lower survival rate was found in IDH1 WT rats (16.67%) compared to BCAT1 knockdown rats (100%) (Figure 2). Thus, we believe that BCAT1 expression level can be used for the potential marker for diagnostic and prognostic assessment in patients with IDH1 WT GBM.

Technically, many different methods have been developed to noninvasively image GBM vasculature [34–37]. Consequently, DSC MRI allows the calculation of physiological parameters such as blood volume, blood-to-tissue transfer constant, and blood-brain-barrier integrity of blood flow. In addition, this implementation can measure the size and the density of tumor vessels and offer additional important information [38–40]. For example, perfusion MRI can help address the issues relating to conflicting treatment effects and tumor vascularity observed with conventional MRI [38]. Therefore, it is important to establish a method that might allow continuous monitoring of treatment response and/or recurrence in GBM patients using DSC perfusion MRI. In this context, we have directly addressed the effects of bevacizumab on tumor vessels, tumor growth, and tumor cell viability using DSC perfusion MRI. Interestingly, immunohistochemical findings, including cell proliferation, vascularity and HIF-1α, showed good agreement with DSC perfusion MRI results (Figure 4).

One limitation of present study should be mentioned; we could not apply the BCAT1 suppression treatment in animal GBMs to verify the BCAT1-related bevacizumab resistance, although we obtained some promising results from animal studies. So, we believe that a future clinical study is warranted.

In summary, the activity of BCAT1 is associated with markers of anatomical, physiological and biochemical angiogenesis and proliferation, as well as of resistance to anti-angiogenic therapy. Thus, BCAT1 knockdown makes the tumors more susceptible to treatment with bevacizumab. In addition, the investigation of bevacizumab resistance increased by BCAT1 in IDH1 WT GBM can be non-invasively assessed by DSC perfusion MRI, which is commonly observed during treatment. These latter effects are of major clinical importance with regard to the early detection and therapeutic response monitoring of IDH1 WT GBM patients. From these observations, we believe that this study could be promising to guide appropriate treatment selection and therapeutic monitoring of IDH1 WT GBM.

## MATERIALS AND METHODS

### Cell lines

Human U87 MG glioma cells were obtained from the American Type Culture Collection (ATCC) and maintained in RPMI medium with 10% FBS at 37°C. The GFP tagged IDH1 WT (GenBank accession number NM_005896) expressing U87 MG cell line was established as previously described [41].

### Virus production and transduction

The sequence verified shRNA lentivectors were produced by the GenTarget Institute. Briefly, shRNA lentivectors were co-transfected with lentiviral packaging plasmids (Cat#: HT-pack) into the lentivirus production cell line (Cat#: TLV-C). The lentivirus was packaged in DMEM medium with 10% serum following GenTarget's virus production protocols. The virus titers were measured via fluorescent cell sorting after particles transducted upon HT1080 cells.

BCAT1 knockdown cells were established by infecting GFP-tagged IDH1 WT-expressing U87 MG with pLenti-U6-shBCAT1-Rsv-RFP-Bsd lentiviral particles (construction summarized in Supplementary Figure S1) for 24 hours in the presence of 4–8 μg/mL polybrene and selected using blasticidin and sorted by FACS. Quantification of knockdown was assessed by western blot analysis and shBCAT1 #1 was the most effective (Supplementary Figure S2).

### Animal model

The study was approved by the institutional animal care and use committee of Seoul National University Hospital. Rat GBM models were used for all experiments except *in vivo* invasiveness assessment, which was performed with mouse GBM model.

### *In vivo* bevacizumab response study in a rat GBM model

We examined 19 male athymic nude rats (mean weight, 250 g ± 30) for orthotopic brain tumor models. The 6-week-old rats were anesthetized with a mixture of zolazepam and xylazine and were placed in a stereotaxic device. A total 3 × 10^6^ IDH1 WT, BCAT1 sh#1 or BCAT1 sh#3 expressing U87 MG glioma cells (IDH1 WT; *n* = 7, IDH1 WT + BCAT1 sh#1; *n* = 7, and IDH1 WT + BCAT1 sh#3; *n* = 4, respectively) was inoculated into the right caudate-putamen region. The cells were injected in the brain using a Hamilton syringe fitted with a 28-gauge needle, which was positioned with a syringe attachment fitted to the stereotaxic device. The following coordinate was used (in mm posterior, lateral and dorsal to the bregma): CPu (0, 1.4, 3.0).

**Figure 5:**
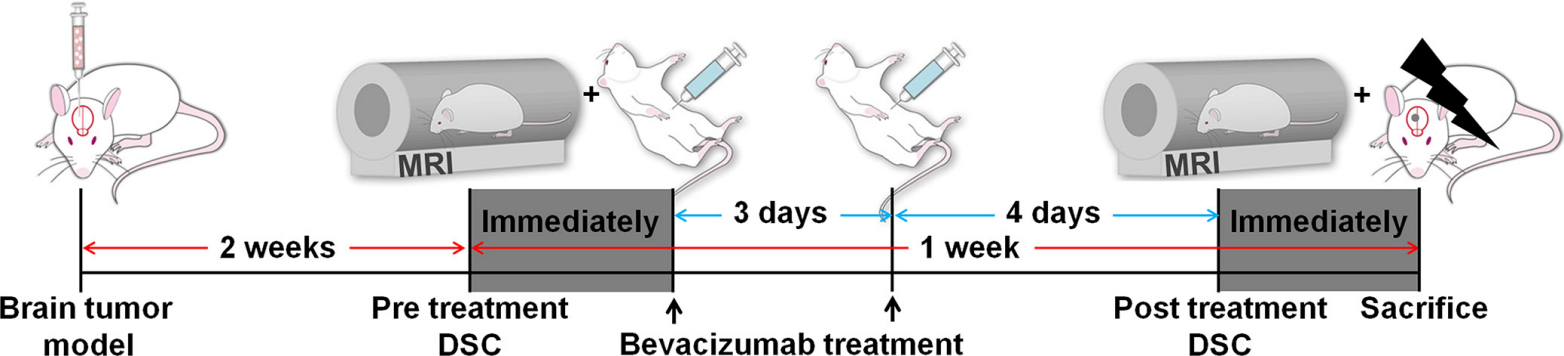
Experimental design for *in vivo* MR study Two groups of rats were intracranially injected with IDH1 WT (*n* = 7) or BCAT1 sh#1 (*n* = 7) human GBM cell lines. After 2 weeks, a pre-treatment DSC perfusion MRI was performed. Bevacizumab (20 mg/kg) was intraperitoneally injected immediately and 3 days after the pre-treatment DSC perfusion MRI. After 7 days, a post-treatment DSC perfusion MRI was performed, and the brains were immediately removed for histological analysis. DSC: dynamic susceptibility contrast.

An experimental design for the *in vivo* study is shown in Figure 5. Two weeks after cranial implantation, pre-treatment DSC perfusion MRI was performed. Then, all rats were treated with an intra-peritoneal injection of bevacizumab (20 mg/kg) twice for a week. Then, the brains were removed for the histological analysis after post-treatment DSC perfusion MRI acquisition.

### MRI protocol

For the *in vivo* animal MR study, the tail vein was catheterized after anesthesia with 1.5–2% isoflurane/oxygen (v/v), and the animals were placed in the 9.4T MR scanner (Agilent Technologies). Throughout each imaging session, animals were wrapped in a warm water blanket and oxygen saturation and heart rate were monitored. A rapid 72 volume coil was used to transmit radiofrequency, and a rapid 72 rat brain coil, placed on the animal's head, was used to receive radiofrequency. The coils were actively decoupled. First, unenhanced anatomic T2-weighted images (repetition time msec/echo time msec = 2000/45, 31 × 35 mm field of view, 256 × 256 matrix, and 12 contiguous 1-mm-thick sections) were obtained in the coronal planes. Then, T2*-weighted DSC perfusion MRI were acquired using a gradient-echo pulse sequence with the following parameters: 25/5, 10° flip angle, 31 × 35 mm field of view, 128 × 96 matrix, three contiguous 2-mm-thick sections, and total acquisition time 1 minute 30 seconds. After an initial ~30-second baseline acquisition, rapid administration of gadoterate meglumine (Dotarem^®^, Guerbet) at a concentration of 0.1 mmol per kilogram of body weight was performed via the tail vein catheter using a syringe pump (Harvard Apparatus) at a rate of 1 mL/min, followed immediately by a 1 mL saline flush at the same injection rate.

### Image post-processing and data analysis

DSC perfusion MRI for *in vivo* animal was processed using commercialized software (Nordic ICE, NordicNeuroLab), in which the CE-T1WI and T2WI were used for structural imaging. The rCBV maps were generated with established tracer kinetic models applied to the first-pass data [15]. To reduce the recirculation effects, the ΔR2* (1/T2*) curves were fitted to a gamma-variate function, which is an approximation of the first pass response as it would appear in the absence of recirculation or leakage. The dynamic curves were mathematically corrected to reduce contrast-agent leakage effects [19]. Normalization of rCBV maps was automatically performed using the mean value of the blood volume outside the tumor, without any intervention of observers. The normalized rCBV (nCBV) maps were presented as color overlays on structural images.

One investigator (S.H.C., 14 years of experience in neuroradiology), who was blinded to the experimental and clinical data, drew ROIs that contained the entire tumor on every continuous section of the co-registered images. Tumor boundaries were defined with reference to the high-signal intensity areas thought to represent tumor tissue on the T2WI. Areas of necrosis, hemorrhage, or macro-vessels were first identified on the conventional MRI sequences and were excluded carefully from the ROIs. After obtaining the total voxel values of the nCBV of each tumor, total volume and mean nCBV of each tumor were calculated. In addition, the tumor volume and nCBV ratio (post-bevacizumab treatment value/pre-bevacizumab treatment value × 100) were also calculated.

### MRS protocol

MRS studies were conducted on a 9.4T MR scanner (Agilent Technologies) 2 weeks after the cell implantation (*n* = 4). Prior to MRS data collection, T2-weighted scout images were acquired for all three orthogonal directions. Using a SPECIAL sequence (repetition time (TR)/echo time (TE) = 4000/2.83 ms, spectral bandwidth (BW) = 5 kHz, 2048 data points), water-suppressed MRS data were collected from the tumor region of the brain for each animal (256 signal averages). Voxel volumes ranged from 11 to 27 mm^3^ [42–46]. Subsequently, water-unsuppressed MRS data were collected with 32 signal averages. To account for potential differences in spectral baseline among the animals, metabolite-nulled baseline spectra were also acquired for all animals with a SPECIAL sequence modified for double inversion (HS10 pulses, BW = 6.6 KHz, duration = 3 ms). The sequence parameters were TI1(1st inversion time)/TI2/TR = 2150/680/4650 ms and 320 signal averages. The rest of the sequence parameters were identical to those used for the metabolite quantification.

Data were processed using MRUI. Data were zero-filled to 4096 points and, after Fourier transformation, line-broadened and phase-corrected. Residual water signals in water-suppressed data were removed by the HLSVD filter. The spectral basis set, consisting of a total of 19 metabolites, was created by referring to the chemical shifts and J–coupling constants of the metabolites. Based on the metabolite-nulled spectra, baseline spectra were modeled for each animal and included in the basis set for spectral fitting. The QUEST time domain fitting method was used for metabolite quantification. Peaks were normalized to the water signal estimated from the water-unsuppressed data.

### Immunohistochemical staining

Immunohistochemical staining was performed using formalin-fixed paraffin-embedded tumor blocks. Briefly, 4-μm-thick tissue sections were deparaffinized in xylene and hydrated by immersing them in a series of graded ethanols. Antigen retrieval was performed in a microwave by placing the sections in epitope retrieval solution (0.01 M citrate buffer, pH 6.0) for 20 minutes; endogenous peroxidase was inhibited by immersing the sections in 0.3% hydrogen peroxide for 10 minutes. Sections were then incubated with the primary mouse monoclonal antibody to BCAT1 (BD Biosciences), mouse monoclonal antibody to human IDH1 R132H (Dianova), mouse monoclonal antibody to human KI67 (UM800033, ORIGENE), goat polyclonal antibody to rat CD34 (R&D Systems), or mouse monoclonal antibody to human HIF-1 alpha (Thermo) in Dako REAL antibody diluent (Dako). Staining for the detection of the bound antibody was evaluated by DAB.

### Statistical analysis

All statistical analyses were performed using two commercial software programs (MedCalc version 13.1.0.0, MedCalc Software). A *p* value < 0.05 was considered statistically significant. Kolmogorov-Smirnov's test was used to determine whether the non-categorical variables were normally distributed. Non-parametric data are presented as median and interquartile range (IQR, range from the 25th to the 75th percentile), and parametric data are shown as the mean ± standard deviation. Based on the results of Kolmogorov-Smirnov's test, an unpaired Student *t*-test or a Mann-Whitney *U*-test was performed, as appropriate, to compare the values between two groups. Kaplan-Meier curves and the log-rank test were used to compare survival times among the groups. A one-way analysis of variance (ANOVA) or the Kruskal-Wallis test with post hoc analysis was performed for the multiple comparisons of the values.

## SUPPLEMENTARY MATERIALS


