# Supplementary material for: Assessment of bevacizumab resistance increased by expression of BCAT1 in IDH1 wild-type glioblastoma: application of DSC perfusion MR imaging
Source: Oncotarget. 2016 Sep 8;7(43):69606–15. doi: 10.18632/oncotarget.11901 (PMC5342501; doi:10.18632/oncotarget.11901)
Supplement: Supplementary file 1 [file oncotarget-07-69606-s001.pdf]

Assessment of bevacizumab resistance increased by expression of BCAT1 in IDH1 wild-type glioblastoma: application of DSC perfusion MR imaging

Supplementary Materials

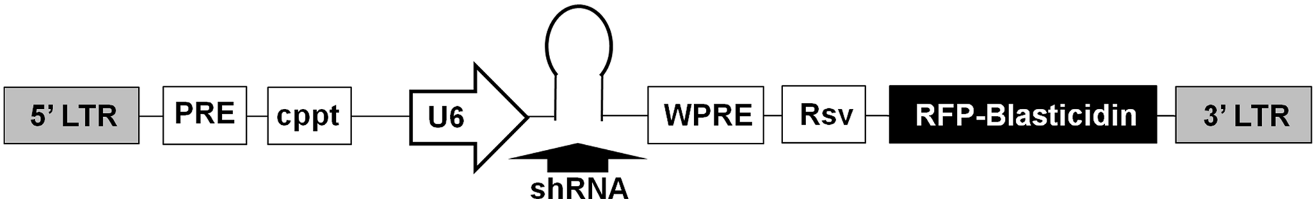

Supplementary Figure S1: Construction of lentiviral vectors. To generate shRNA-expressing lentiviral constructs, shRNA sequences were PCR amplified and cloned into GenTarget's lentiviral shRNA expression vector (pLenti-U6-shRNA-Rsv-RFP-Puro) according to the product protocols. A RFP-Blasticidin (RFP-Bsd) fusion dual marker was expressed under an RSV promoter, and the shRNA was expressed under enhanced constitutive human U6 promoter in the vector.

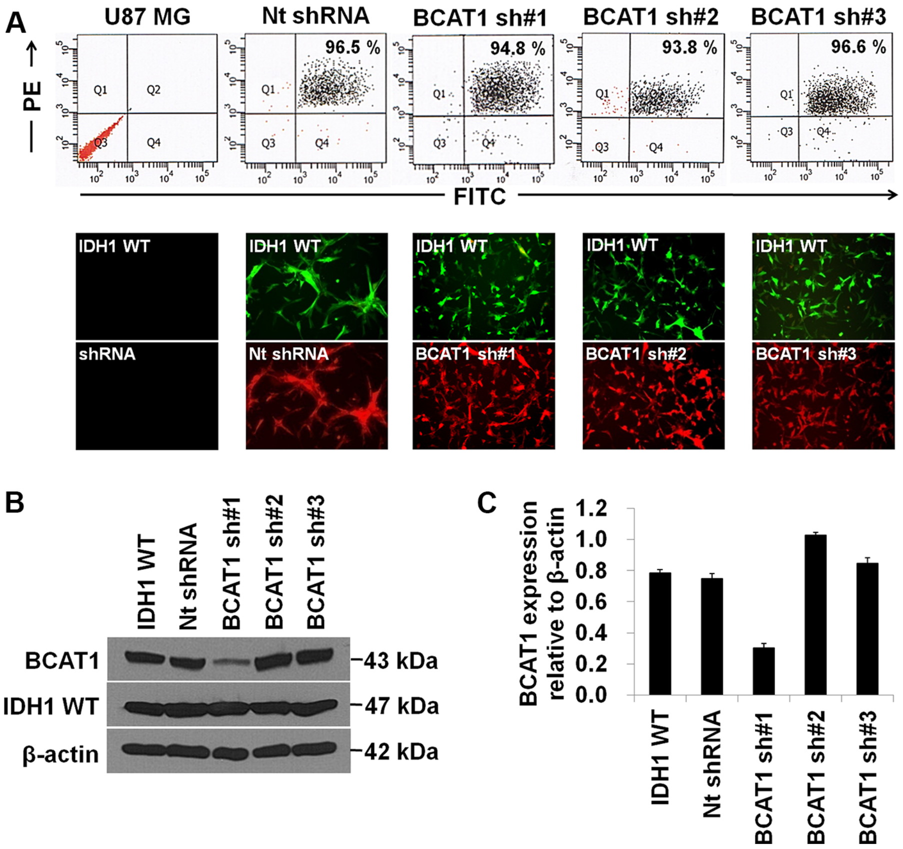

Supplementary Figure S2: Establishment, selection of BCAT1 knockdown cells. pLenti-U6-shBCAT1 #1 (GCTCATCAGCT TTGCACTATG), #2 (ATCTGCTAGTCTGTATATTCG), #3 (GGGAATTACGGCTCATCTCTT) were used to generate the lentiviral vectors containing human BCAT1 targeting sequences. Approximately 93% of cells expressed IDH1 WT and shBCAT1 simultaneously. The expressing level was analyzed by western blot, and BCAT1 was most effectively knocked down by the sequence BCAT1 sh#1.

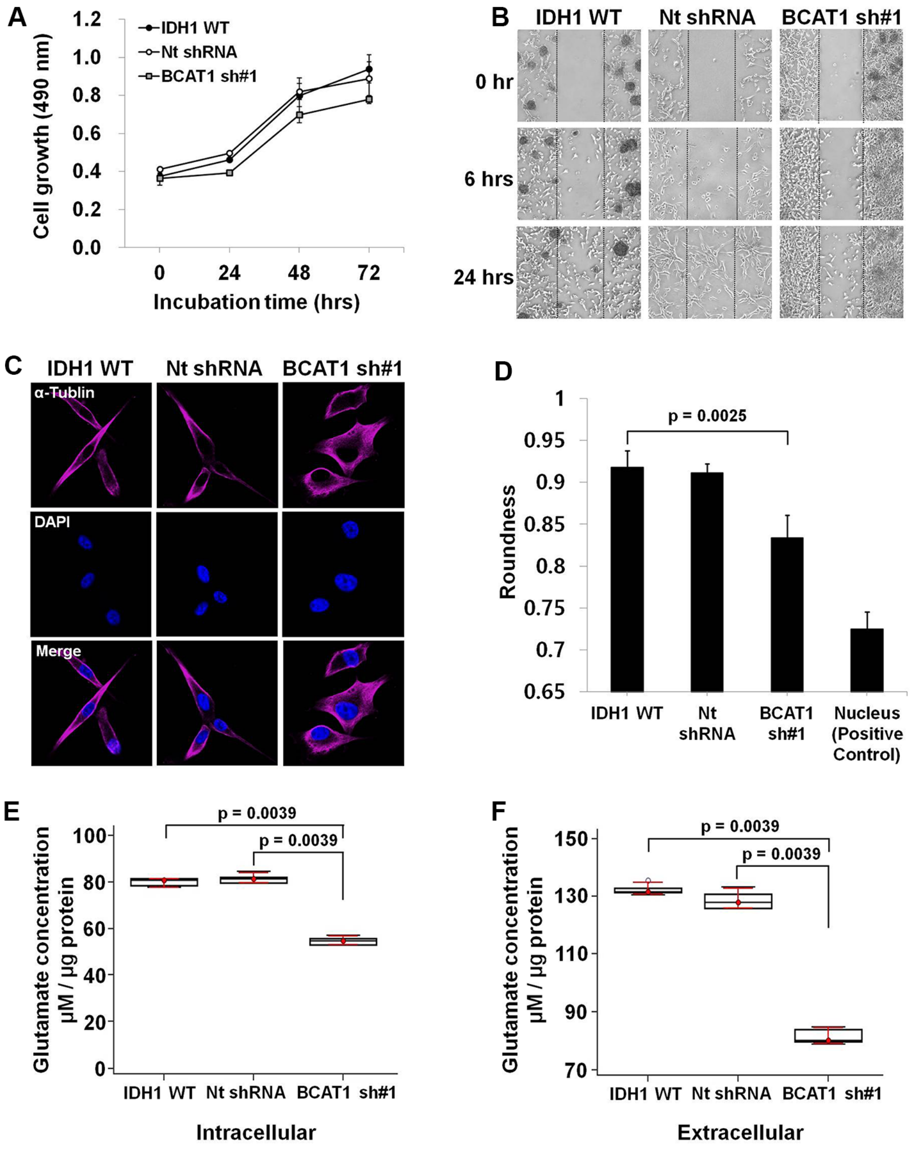

Supplementary Figure S3: Characterization of cells. (**A**) Cell proliferation of IDH1 WT, BCAT1 sh#1 and their corresponding control cells grown in RPMI medium with 10% FBS for the indicated times. BCAT1 sh#1 suppresses the cell growth. (**B**) The wound healing by migrated cells at 0, 6, and 24 hours is imaged. IDH1 WT cells with BCAT1 sh#1 have a significantly reduced motility and migration compare to IDH1 WT cells. (**C**) Cell morphology investigated by immunofluorescence staining. BCAT1 sh#1 cells are reduced in the extensions and changed to a round shape compared with IDH1 WT (Tubulin: purple, Nuclear: blue). Moreover, (**D**) Roundness of BCAT1 sh#1 cells was significantly different with IDH1 WT. The round shape is approximately near to 0.5 and used nucleus as positive control ($p = 0.0025$). (**E**) Intracellular and (**F**) extracellular glutamate concentration of indicated cell lines. Released glutamate was decreased in BCAT1 sh#1 cells in both intra and extracellular samples.

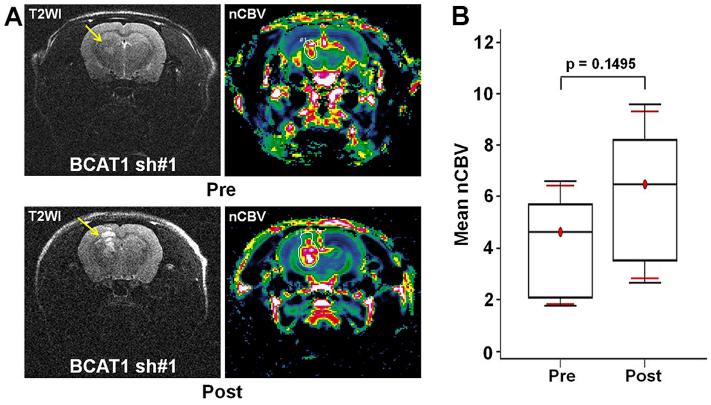

Supplementary Figure S4: *In vivo* DSC perfusion MRI of BCAT1 sh#1 rats without bevacizumab treatment. Post-treatment DSC perfusion MRI showed that BCAT1 sh#1 rats ($n = 4$) injected with normal saline, instead of bevacizumab, increased in nCBV compared with pre-treatment MRI. (4.63 [IQR, 2.10–5.71] vs 6.50 [3.53–8.21]; $p = 0.1495$) Yellow arrow in T2WI indicates the tumor.

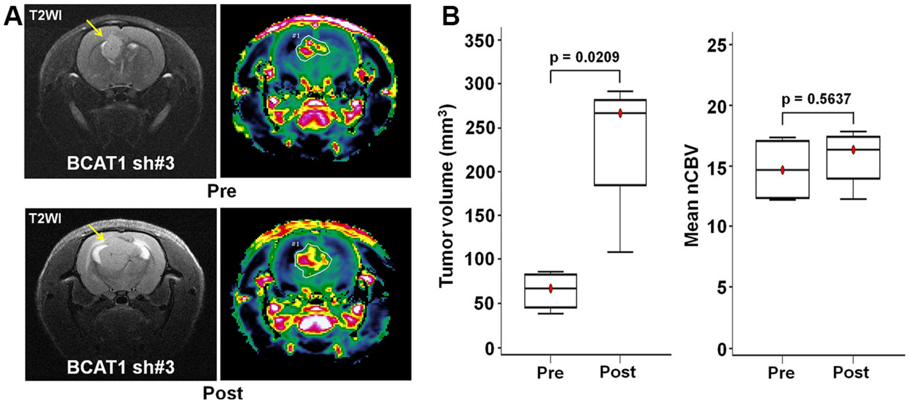

Supplementary Figure S5: *In vivo* DSC perfusion MRI of BCAT1 sh#3 rats. For the evaluation of off-target effects, we performed DSC perfusion MRI in BCAT1 sh#3 rats ($n = 4$) before and after bevacizumab treatment, which revealed similar change to IDH WT tumors in terms of both tumor volume (66.96 mm^3 [IQR, 46.24–82.62] vs 267.04 mm^3 [184.97–282.20]; $p = 0.0209$) and nCBV (14.71 [IQR, 12.39–17.08] vs 16.34 [13.96–17.41]; $p = 0.5637$). Yellow arrow in T2WI indicates the tumor.

MATERIALS AND METHODS

Fluorescence activated cell-sorting analysis

GFP-tagged IDH1 WT and RFP-tagged shBCAT1 in transduced cells were analyzed using a FACSCalibur cell sorter (BD Bioscience) with Cell-Quest software (BD Bioscience). A 488 nm laser for GFP and a 561 nm laser for RFP were used for excitation. GFP and RFP were detected using 530/40 nm and 630/75 nm bandpass filters, respectively. The sorted cells were visualized by fluorescent inverted microscope (Olympus, IX51) and used for *in vitro* and *in vivo* studies.

Western blot analysis

The protein levels were evaluated by western blot analysis. Cells were lysed in ice-cold lysis buffer (Cell Signaling), and the concentration of protein was evaluated with the bicinchoninic acid method (Pierce Biotechnology). Approximately 30 μg of protein were loaded in each lane of a polyacrylamide denaturing gel for electrophoresis. After electrophoresis, the protein was transferred to nitrocellulose membranes for blotting. We used a rat monoclonal antibody to human IDH1 (Dianova), a mouse monoclonal antibody to human BCAT1 (OriGene), and a rabbit polyclonal antibody to β-actin (Abcam). Primary antibodies were detected by horseradish peroxidase-conjugated antibodies (Santa Cruz Biotechnology).

Proliferation assay

For assessment, the three groups of cells (IDH1 WT and Nt shRNA, BCAT1 sh#1) were seeded on 96-well plates at an initial density of 5×10^4 cells/well, and proliferation was measured 24, 48, and 72 hours after plating by MTT assay. Briefly, 10 μL of 5 mg/mL MTT agent (Sigma) was added and incubated for 2 hours. Then, the medium was gently aspirated, and 150 μL DMSO was added and incubated for 15 minutes. The plates were read on a microplate reader using a test wavelength of 490 nm.

Migration assay

The analysis of cell motility and migration was carried out using Culture-Inserts ready to use in a μ-Dish 35 mm (ibiTreat, item #: 81176, IBIDI), which allowed the use of high-resolution microscopy in a 35 mm Petri-dish with 12 mm walls. [1] Cells were seeded in RPMI, and when cells reached 100% confluence, the inserts, which contain 500 μM gaps, were taken out. Cells were then grown and allowed to migrate for 48 hours. Cell images were collected using a microscope (Leica DMIL LED) equipped with a 5× objective.

Immunofluorescence staining

Cells were seeded on glass coverslips. The next day, cells were fixed in 4% formaldehyde, rinsed three times in PBS and permeabilized in PBS containing 0.1% Triton X-100. After rinsing with PBS, cells were incubated in 3% BSA for 30 minutes at room temperature for blocking. Samples were incubated for 1 hour with the primary antibody (mouse antibody to α-tubulin, Sigma) and for 1 hour at room temperature with the secondary antibody (Cy5-conjugated goat mouse-specific antibody, Life Technology) after mounting with DAPI. For fluorescence imaging, images were taken using a confocal microscope (Zeiss LSM 510 META) and cell roundness was quantitatively analyzed by using MetaMorph® Image Analysis software, where the value ranges 0.5 – 1, and the value of 0.5 means completely round in shape.

In vitro glutamate quantification at the cellular level

Cells were cultured for 48 hours in a medium without phenol red, the medium was collected, and the cells were lysed with lysis buffer (Cell Signaling). Glutamate concentrations in the medium and in the cell lysate were determined with the glutamine/glutamate determination kit (GLN-1; Sigma) according to the manufacturer's instructions. The amount of NADH is proportional to the amount of glutamate and was measured six times using a spectrophotometer at 340 nm. The data were normalized to the protein level [2, 3].

REFERENCES

1. Li F, Yu X, Szynkarski CK, Meng C, Zhou B, Barhoumi R, White RE, Heaps CL, Stallone JN, Han G. Activation of GPER Induces Differentiation and Inhibition of Coronary Artery Smooth Muscle Cell Proliferation. PloS one. 2013; 8:e64771.
2. Tonjes M, Barbus S, Park YJ, Wang W, Schlotter M, Lindroth AM, Pleier SV, Bai AH, Karra D, Piro RM, Felsberg J, Addington A, Lemke D, et al. BCAT1 promotes cell proliferation through amino acid catabolism in gliomas carrying wild-type IDH1. Nature medicine. 2013; 19:901–908.
3. Kung HN, Marks JR, Chi JT. Glutamine synthetase is a genetic determinant of cell type-specific glutamine independence in breast epithelia. PLoS genetics. 2011; 7:e1002229.
